# The serious mental illness health improvement profile [HIP]: study protocol for a cluster randomised controlled trial

**DOI:** 10.1186/1745-6215-12-167

**Published:** 2011-07-04

**Authors:** Jacquie White, Richard J Gray, Louise Swift, Garry R Barton, Martin Jones

**Affiliations:** 1 Faculty of Health and Social Care, University of Hull, Hull, HU6 7RX, UK; 2 Faculty of Health, University of East Anglia, Norwich, NR4 7TJ, UK; 3 Faculty of Medicine and Health Sciences, University of East Anglia, Norwich, NR4 7TJ, UK; 4 Surrey and Borders NHS Foundation Trust and the University of Surrey, UK

## Abstract

**Background:**

The serious mental illness Health Improvement Profile [HIP] is a brief pragmatic tool, which enables mental health nurses to work together with patients to screen physical health and take evidence-based action when variables are identified to be at risk. Piloting has demonstrated clinical utility and acceptability.

**Methods/Design:**

A single blind parallel group cluster randomised controlled trial with secondary economic analysis and process observation. Unit of randomisation: mental health nurses [MHNs] working in adult community mental health teams across two NHS Trusts. Subjects: Patients over 18 years with a diagnosis of schizophrenia, schizoaffective or bipolar disorder on the caseload of participating MHNs. Primary objective: To determine the effects of the HIP programme on patients' physical wellbeing assessed by the physical component score of the Medical Outcome Study (MOS) 36 Item Short Form Health Survey version 2 [SF-36v2]. Secondary objectives: To determine the effects of the HIP programme on: cost effectiveness, mental wellbeing, cardiovascular risk, physical health care attitudes and knowledge of MHNs and to determine the acceptability of the HIP Programme in the NHS. Consented nurses (and patients) will be randomised to receive the HIP Programme or treatment as usual. Outcomes will be measured at baseline and 12 months with a process observation after 12 months to include evaluation of patients' and professionals' experience and observation of any effect on care plans and primary-secondary care interface communication. Outcomes will be analysed on an intention-to-treat (ITT) basis.

**Discussion:**

The results of the trial and process observation will provide information about the effectiveness of the HIP Programme in supporting MHNs to address physical comorbidity in serious mental illness. Given the current unacceptable prevalence of physical comorbidity and mortality in the serious mental illness population, it is hoped the HIP trial will provide a timely contribution to evidence on organisation and delivery of care for patients, clinicians and policy makers.

**Trial Registration:**

ISRCTN: ISRCTN41137900

## Background

### Serious mental illness and physical comorbidity

Improving the physical health of people with serious mental illness [SMI] (people with a diagnosis of schizophrenia, schizoaffective or bipolar disorder) is an important public health challenge[1,2]. Comorbid physical illness dramatically reduces life expectancy; epidemiological studies report 20-25 years earlier mortality in schizophrenia and 10-15 years in bipolar disorder [3,4]. Metabolic disorders such as diabetes, hyperlipidemia and hypertension are highly prevalent, exceeding 50% in some studies[5]. Cardiovascular disease [CVD] is the most common cause of early mortality; lifestyle and risk factors are common and may be exacerbated by antipsychotic medication[6,7]. Rates of respiratory disease, HIV and some cancers are higher than expected[8]. Poor eye, foot, bowel and dental health, sleep problems and sexual dissatisfaction contribute to social exclusion[9-11].

### Screening and intervention

The physical health needs of SMI patients has long been overlooked by both primary and secondary care [12-14] prompting a number of guidelines (e.g. [15-18]. The first step is monitoring but randomised controlled trial evidence to support a robust method in this population is lacking[19].

Although considerably more likely than the general population to visit their GP, 2% SMI patients had had their cholesterol checked and recorded in one study[20]. In 606 inpatients, 18% had their weight recorded and 4% their cholesterol checked during admission[21]. Data from 1,966 outpatients revealed 11% had documented results for the four tests that should be undertaken each year for those at risk of metabolic syndrome[22]. No formal provision to record, share and use information from physical health screening in the Care Programme Approach has been reported by 28% of Local Implementation Teams[23]. The Department of Health [DH] has encouraged primary and secondary services to work together to improve physical health outcomes and adopt local care pathways, recommending a physical health 'wellbeing programme' [WBP][1]. The WBP, facilitated by nurse advisors trained by key opinion leaders and funded by the pharmaceutical industry, enabled some NHS Trusts to set up physical health clinics at pilot sites, almost exclusively focusing on identifying and reducing modifiable cardiovascular risk factors. Wider dissemination of the programme was by a cascade of training from the nurse advisors who were then withdrawn. Data from one large mental health [MH] Trust indicate that this approach diluted fidelity to the original WBP model with fewer sessions offered to patients and no replication of the positive results seen[24].

Mental Health Nurses [MHNs] are well placed to profile SMI patients' physical health needs, plan and facilitate evidence based care and communicate with primary care colleagues[8] but there is poor preparation for this role[25] with half reporting no previous training in one survey[26]. Relatively short courses can be effective in enabling MHNs to acquire competence and confidence in new ways of working that improve clinical outcomes[27,28]. Two small studies have shown the potential to improve MHNs' detection of physical health problems following brief training[29,30].

### The serious mental illness Health Improvement Profile [HIP]

The HIP is a 27-item gender specific profiling tool that enables MHNs and patients to work together to identify and 'red flag' aspects of physical health. Importantly the HIP is a change tool, not merely a detection tool. It directs the nurse and patient to select the action to take next and provides a template for communicating with psychiatrists and general practitioners [GPs]. The HIP has been designed to fit on one side of A4 paper and has 5 columns indicating the variable at risk for assessment (e.g. smoking status), level (result), Green (e.g. 'non smoker'), Red (e.g. 'passive smoker/smoker') and the recommended action for red group (e.g. advice that all smoking is associated with health risks, refer to NHS smoking cessation service). The HIP should be completed at least annually, the recommended frequency of screening for patients with SMI[17,18].

A series of literature reviews established the variables at risk in SMI, normal and abnormal ranges and recommended action informing the development of the HIP through piloting stages[8,31]. Utility and acceptability was demonstrated by evaluation of an exploratory case series where patients made lifestyle changes as a result of completing the HIP with their nurse and GPs and psychiatrists altered treatment plans[32]. Training to facilitate the HIP takes between 3 and 6 hours. The 6-hour training package includes how to use the HIP, how to engage patients in health behaviour change and support to develop a patient specific health action plan. A HIP manual is provided. It is unknown if the HIP Programme is more effective in improving physical wellbeing in patients with SMI after a year than current practice in this population so a randomised controlled trial has been recommended[19].

## Methods/design

The study is aimed at people living with SMI in the community (defined as those with a diagnosis of schizophrenia, schizoaffective or bipolar disorder) and will investigate the impact of the HIP and the brief HIP training package for nurses [HIP Programme] in patients with SMI on the caseload of community Mental Health Nurses [MHNs]. Registered MHNs working in a community mental health setting will be recruited from adult community mental health, assertive outreach and recovery teams serving the urban, coastal and rural communities of Norfolk, North Suffolk and Lincolnshire. Once the MHN enters the trial, eligible SMI patients will be identified from their caseload and invited to participate. A cluster randomised controlled design with the nurse as the unit of randomisation has been selected because our aim is to improve patient outcomes by an intervention directed at the level of the nurse (the HIP Programme). We have planned the study in accordance with the Consolidated Standards of Reporting Trials [CONSORT] cluster trial reporting extension standards[33].

### Objectives

The primary objective is to determine the effects of the HIP programme on patients' physical wellbeing over 12 months compared to treatment as usual (objective 1).

The burden of physical comorbidity in patients with SMI is likely to incur additional service costs in terms of diagnostic screening and treatment of previously unrecognised or untreated comorbidity. An economic evaluation will assess if the HIP Programme represents a cost-effective use of scarce NHS resources (objective 2). Impact of the HIP Programme on mental health related quality of life (objective 3) and the physical health care attitudes and knowledge of MHN (objective 4) will be evaluated. In addition to the main trial, any modification of cardiovascular risk factors by patients (objective 5) and evidence of acceptability of the HIP Programme to the NHS (objective 6) will be explored in the HIP Programme group. There will, therefore, be two parts to the study:

### Part 1

#### Impact and cost effectiveness (Objectives 1-3)

**Figure 1:**
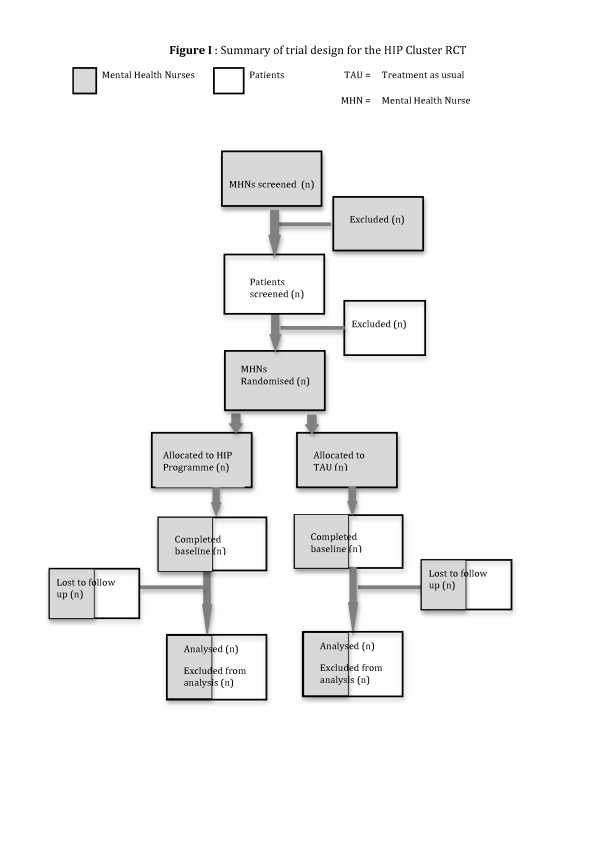
**Summary of trial design for the HIP Cluster RCT**.

A 2-arm single-blind, parallel group randomised controlled trial design (clustered at the level of the MHN). MHNs will be randomised to either the HIP Programme group or treatment as usual [TAU]. Figure 1 summarises the trial design. For details of inclusion and exclusion criteria of MHNs please see pages 12-13.

#### Physical health care attitude and knowledge of MHNs (Objective 4)

MHNs attitude towards and knowledge for a physical health care role will be measured at baseline and at 52 weeks in both groups using an online survey.

#### Cardiovascular risk (Objective 5)

Cardiovascular risk factors will be measured using a 'within treatment' group design in the HIP Programme group patients at baseline and at 52 weeks to allow before-after change to be estimated (Objective 5).

### Part 2

#### Acceptability of the HIP Programme in the NHS (Objective 6)

To assess acceptability of the HIP Programme in the NHS (objective 6), a process observation will be conducted in the HIP Programme group. A retrospective documentation audit of the secondary care patient record will capture details of recorded physical health needs, care plan interventions, goals, review dates and any communication across the primary-secondary care interface. Perceptions in a sample of MHNs, patients, their psychiatrists and GPs will be explored and analysed using a semi structured interview or focus group design.

### Outcome measures

#### Part 1: Cluster RCT (Objectives 1-5)

The Medical Outcome Study (MOS) 36 Item Short Form Health Survey version 2 [SF-36v2] is a self-report multidimensional measure of health-related quality of life and wellbeing with well established psychometric properties[34]. The revised version of the SF36 has improvements in item wording and format and a 6 fold increase in the range of scores produced. Participant burden is not increased[35]. The scales of the SF-36v2 address eight health domains and two summary measures are provided: a physical component summary score [PCS)], and a mental component summary score [MCS]. The PCS was selected as the primary quality of life (QoL) outcome measure as it has been shown to have good sensitivity to change in a population of outpatients with schizophrenia [36]We acknowledge that, at least theoretically, mental health-related quality of life may change as a result of implementing the HIP because patients will become aware of previously unknown morbidity so the MCS will also be used.

In line with a previous economic evaluation for patients with schizophrenia[37], levels of health care resource use [HRU] will be captured via an amended version of the Client Services Receipt Inventory[38]. This patient self-report measure (HRU (Patient)) will monitor health professional visits, hospital admissions, medication, etc. and be administered to all study participants at baseline and at the 52 weeks post randomisation point. Additionally, nurses will be asked to report the time taken to complete the HIP, referrals made, medication changes etc. every time the HIP is used (HRU (Nurse)).

The EuroQol 5 Dimension questionnaire [EQ-5D] is an established, standardised health-related quality of life instrument used extensively in clinical studies[39]. It provides a simple descriptive profile of each respondent and a single index value for their perceived current health status. It comprises five items covering the domains of mobility, self-care, usual activity, pain/discomfort and anxiety/depression and a visual analogue scale. The EQ-5D is cognitively simple, taking only a few minutes to complete. Instructions to respondents are included in the questionnaire. It will be administered to all patient participants in the study at baseline and at the 52 weeks post randomisation visit.

The Mental Health Nurse Physical Health Attitude Scale [PHASe] is a 29 item questionnaire with established validity, designed to capture MHNs attitude towards their physical health care role [40]. It does not evaluate knowledge so it has been adapted to include 20 physical health care multiple choice questions [MCQ]. All MHNs in the study will be invited to complete the adapted PHASe at baseline once randomised and then again at the end of a year via a secure website.

QRISK^®^2 is a cardiovascular disease prediction algorithm providing an individualised estimate of risk using cholesterol, blood pressure and body mass index values, medical history and taking account of the independent contributions of ethnicity and social deprivation in the UK (by post code). Data on the required variables will be extracted from the HIP and the Patient Baseline Demographics Form for all subjects in the HIP Programme Group allowing an individual QRISK^®^2 to be calculated. Face validity, good discrimination and calibration of items has been established for QRISK^®^2 [31,41].

### Part 2: Process observation (objective 6)

A HIP Audit Form will capture details of recorded physical health needs, care plan interventions, goals, review dates and any communication across the primary-secondary care interface (e.g. letters to and from GPs, practice nurses) from the secondary (mental health) care patient record. It will be administered in a standardised way by the Trial Coordinator to the records of a purposeful sample of 36 HIP Programme Patient Participants. One to one interviews (patients), focus groups (nurses) and telephone interviews (psychiatrists and GPs) will be facilitated by the Trial Coordinator using a series of open-ended questions with the aim of:

• Obtaining insights into the patients' and health professionals' experiences of using the HIP

• Considering which elements of the HIP Programme were perceived as being most and least helpful

• Exploring the participants' perceptions of the effect that they think the HIP Programme [as opposed to TAU] has on them

• Uncovering any potential barriers and roadblocks to using the HIP.

• Exploring how the HIP could be refined and enhanced.

### Recruitment and randomisation

Registered MHNs working in a community mental health setting can be included if they have been registered with the Nursing and Midwifery Council [NMC] for at least 6 months, employed at Agenda for Change band 5-7, work in one of the community mental health teams across the two Trusts' localities and have at least 5 patients on their caseload with a primary diagnosis of SMI (as confirmed by their Team Leader). Patients can be included if they are aged over 18 years, on the caseload of the MHN participant at the start of the project and have a primary diagnosis of SMI (last recorded diagnosis in the patient record either being schizophrenia, schizoaffective or bipolar disorder as confirmed by the Team Leader).

Following screening, MHNs will be excluded if they are in their period of preceptorship, about to go on maternity leave, are pregnant or up to 6 months post partum. Following screening, patients will be excluded if they currently lack the capacity to consent to treatment as documented by a heath professional on Form 4 in their case notes[42], have a serious or unstable medical condition (e.g. advanced/incurable cancer; severe co-morbidity such as people on renal haemodialysis, end-stage COPD or severe unpredictable pain), are pregnant or 6 months post partum or where the Team Leader considers participation in the trial will put the patient, nurse or member of the research team at increased risk or increased cost to the service to manage risk.

### Part 1

Clinical Community Team Leaders will be asked to nominate MHNs who meet the inclusion criteria to the Research Team who will then invite the MHN to participate by letter. A member of the research team will visit those nurses who respond positively to the invitation, exchange information and take informed consent. This will continue until a total of 50 MHNs have entered the trial. The importance of not sharing the HIP tool or any training material will be explained to all nurses participating in the trial.

Once the MHN is consented, their Team Leader will be asked to list all patients with SMI on their caseload and allocate them a number to maintain anonymity. The Team Leader can justify exclusions in liaison with the Trial Coordinator as necessary. Following random selection of potential patients from this number list by the University of East Anglia Clinical Research and Trials Unit [CRTU], the Team Leader will break the number code and ask nurse participants to hand a letter of invitation and information pack to individual patients. Additional copies of all information will be made available as required (e.g. for carers).

### Part 2

This will start once the first 20 MHNs in the HIP Group have completed Part 1 of the trial and include all patients who remain in the study at one year:

1. UEA CRTU will randomly select a maximum of 2 patients each from each of the MHN study patients until 36 patients are identified for the retrospective case note audit.

2. The Process Observation Lead will select potential subjects for the interviews and focus groups through a process of purposeful sampling using screening information. The Process Observation Lead will send letters requesting an RSVP if able to attend an interview/the focus group date and will purposefully recruit to these groups until 10 patient interviews,10 places in the nurse focus groups per Trust site and 10 telephone interviews (for 5 psychiatrists and 5) GPs are booked. Between 6 and 12 interviews are recommended for qualitative interviews where there is participant homogeneity [36].

#### Informed Consent

Written informed consent will be obtained prior to randomisation for all nurse participants, prior to baseline data collection for all patient participants and prior to the start of any interview or focus group for any patients or nurses who choose to attend. Baseline data collection will take place before the result of random allocation is revealed to any individual participant. A signed consent form will be requested by post from all doctors booking a telephone interview. At each data collection point involving an interview ongoing verbal consent will be sought and participants reminded of their right to withdraw. In addition written information about the purpose of the PHASe (adapted) will be available to read in a separate window before the MHN decides if they wish to participate in the online survey or not. MHNs participating in the online survey will not be required to sign a specific consent form as they can decide if they want to complete the survey or not at each data collection stage. However, they will be asked to indicate they have read and understood the study information before they can submit their responses.

All written information will include contact information (telephone numbers and email) for the project team and the local office of the NHS Patient Advice and Liasion Service [PALS] should any potential participants want further information at any stage to support their decision. Information will also be exchanged about the availability of the same written information or further information for carers, should they wish them to be involved. A copy of the signed Informed Consent form will be given to the participant. The original signed form will be retained at the central Trial Office (University of East Anglia). Where the participant is a patient, an additional copy will be filed in the case record and their GP, Psychiatrist and Care-coordinator will be informed in writing. Patient participants will also be reminded that although data are anonymised, any disclosure which potentially puts them or others at risk will be communicated back to their MHN and Care Co-ordinator (if this is a different person to the nurse). Similarly nurse participants will be reminded that any disclosure or evidence of poor practice will be communicated back to their Team Leader. In both cases the participant will be informed.

### Randomisation

#### Part 1

All consented MHNs will be randomly assigned to either the HIP or TAU arm of the trial. The randomisation schedule will be designed, held and administered by the CRTU. Once the Trial Coordinator receives a signed informed consent form, the MHN will be allocated a unique identification number. Once the MHN is randomised no more patients from their caseload can be included due to the need to complete all baseline measures before the training intervention takes place. The MHN will be randomised once 5 patients have been recruited from their caseload or after 6 weeks, whichever occurs first. At this stage their number will be sent to the CRTU by the Trial Coordinator, where allocation will be by permuted blocks of random size. The Trial Coordinator will be informed of the allocation and will directly contact the MHN to inform them of their group allocation and arrange for them to attend the HIP training if appropriate. This will maintain blinding of the data collectors to group allocation of participants.

All patients on the consented MHN subject's caseload meeting the inclusion criteria are eligible to participate. Once a MHN has given their informed consent (but before they are randomised) the Trial Coordinator will ask the Clinical Team Leader to create a potential list of patients from the caseload, allocating every patient on this list an identification number. The CRTU will then generate a list of 5 first choice and 5 reserve patient numbers at random and letters will go out to these patients via the Team Leader and Nurse Participant inviting them to participate. Interested patients will be asked to contact the research team to arrange a visit from a researcher to enable the informed consent process to be followed. This is to attempt to maintain anonymity of the patient before they make a choice about their interest in taking part and reduces any risk of selection bias.

#### Part 2

The Process Observation Lead, will purposefully select participants for the Process Observation after patients have completed 12 months in the study using information provided at baseline to achieve as representative a sample as possible e.g. diagnosis, nursing grade, GP from small practice or large health centre, urban or city region. 36 patients from the HIP Programme group will be purposefully selected for the HIP Audit. A letter of invitation to the interview or focus group, information sheets and response slips will be sent in the post with a follow up information exchange and informed consent visit from the Trial Coordinator once a positive response is received until 10 participants are identified for each group or set of interviews. Psychiatrists and GPs will not be visited but information exchanged and consent will be checked at the start of the telephone interview.

### Baseline and follow up assessments

Assessments will be completed by a researcher and take place at baseline and 52 weeks post-randomisation. Demographic information will be collected at baseline e.g. grade, caseload size, year of qualification, previous physical health care education (MHNs) and diagnosis, age, post-code (patients). The following validated measures will be used at baseline and 52 weeks in both the HIP and TAU groups:

1. PCS and MCS of the SF36v2

2. EQ-5D

3. HRU (patient)

4. PHASe adapted

The following will be used at baseline and at 52 weeks in the HIP programme group only:

1. HRU: Health Service Use Questionnaire (nurse)

2. The QRISK^®^2

and in a sample of patients from the HIP Programme group after 12 months

1. HIP Audit Form

Feedback will be sought from a sample of HIP Programme group participants in the Patient Interviews, Nurse Focus Groups and Psychiatrist/GP telephone interviews after one year using the Patient Semi-structured Interview Schedule, the Nurse Focus Group Interview Schedule and the Psychiatrist/GP Telephone Interview Schedule.

### Description and core principles of the intervention

The HIP is a gender specific 27-item physical health risk assessment tool. The profile includes items on cardio-vascular risk factors such as BMI (body mass index), cholesterol, diet, smoking and exercise but also addresses other aspects of wellbeing such as sexual health and satisfaction, sleep, dental and eye health and breast, prostate and testicular self examination. Each of the 27 items are flagged either green (healthy) or red (not healthy). The HIP then directs nurses to evidence/guideline-based interventions for each of the red flagged items[43].

HIP training takes place in a 6 hour workshop delivered by JW, RG or MJ and will include: how to use the HIP; how to engage patients in health behaviour change and work with them to develop a patient specific health action plan. A HIP Manual will be provided. Competency in physical observation skills are expected of all MHNs employed in the NHS, assured through a continuing professional development process which will be signposted in the training, the HIP Manual and by the Team Leaders through the usual clinical supervision processes, if necessary.

### Definition of End of Study

#### Part 1

Date of the last 52-week visit for the last patient.

#### Part 2

Date of the last interview/focus group or date of the last patient HIP Audit visit, whichever occurs last.

## Analysis

### Description of Statistical Methods

The analysis and reporting of the trial will be undertaken in accordance with Consolidated Standards of Reporting Trials [CONSORT] [44,45]. Standard methods will be used to provide tabular and graphical summaries as appropriate for continuous and categorical variables. Primary and secondary outcomes will be compared between intervention and control groups using linear or logistic regression as appropriate including a random term to allow for nurse clusters. Further adjusted estimates will be calculated by including the baseline value of the outcome in these models. Where between group differences are observed in demographics or patient characteristics at baseline, terms representing these variables will also be included to obtain adjusted estimates of the intervention effect. The characteristics of the MHNs will be compared between groups. If differences occur these may be incorporated into a multilevel model structure. The latest available versions of the Statistical Package for the Social Sciences [SPSS] and Stata will be used for the analysis. It is not our intention to conduct any subgroup analyses.

### Baseline analyses

To assess external generalisability, demographic and clinical characteristics of patient participants invited to participate in the study at baseline will be compared with participants who are subsequently randomised and participants who are screened but not randomised. The specific criteria by which participants are excluded from randomisation will be tabulated. Baseline comparability will be assessed using descriptive statistics only.

### Number of Participants

#### Part 1

A sample size of 50 MHNs will be sought (25 in the HIP and 25 in the TAU group), with 5 patients each resulting in an overall sample size of 250 patients (125 in the HIP and 125 in the TAU group). Based on a standard deviation of about 12 points as found in MOS patients (adults in various settings with SMI in the US), a difference in means between intervention and TAU of 6 points in the SF36v2 PCS subscale is equivalent to a medium effect size. The proposed sample size gives a power of 86% to detect this if an intraclass correlation of 0.1 is assumed and nearly 80% if an intraclass correlation of 0.2 is assumed using a 2-sided significance level of 0.05 and allowing for an estimated 20% attrition rate i.e. 1 patient per nurse.

All MHNs in the HIP and TAU groups will be invited to participate in the online PHASe adapted at baseline and one year. Response rates will be reported. All patients remaining the HIP Group at one year will provide data for evaluation of any change in their QRISK^®^2 Scores. Retention rates will be reported.

#### Part 2

The patient records of the 36 randomly selected patients from the HIP Programme Group will be audited using the HIP Audit Form. We will purposefully recruit from the HIP Group (on completion of Part 1) 10 patients to participate in the Patient Semi-structured Interviews, 10 nurses to participate in a Focus Group (per Trust study site) 5 psychiatrists and 5 GPs to participate in the telephone interviews. (30 in total). The aim of this element of the project is to elicit general feedback that will help in refining and further developing the HIP. The qualitative literature indicates that this number of data collection interviews per homogenous professional or patient group should allow us to reach saturation of themes [46].

### Feasibility of target sample size

Localities in the two NHS sites provide services via community mental health, assertive outreach and recovery teams. Across the two NHS Trusts there are a total of 16 localities and 33 teams. We will invite every band 5-7 MHN in these teams to participate and once recruited, screen every patient on their caseload for the inclusion criteria before inviting randomly selected patients to take part as a patient subject in the randomised trial. To make this achievable we will work closely with Clinical Team Leaders. Our planned recruitment rate is 25 patients per month (excluding August and December).

### Efficacy analysis

Efficacy of the intervention will be assessed by comparing the patient outcomes at 52 weeks between the two groups allowing for the clustering effect of the nurse. Further, adjusted estimates will be obtained by identifying baseline variables that differ between the two groups, which are related to the outcome and incorporating these into the analyses.

### Inclusion in Analysis

An intention to treat (ITT) analysis will be performed. The ITT analysis set will comprise all patients who were randomised to a group at baseline (HIP Programme or TAU group), irrespective of any change to the other group over the course of the trial. This is the main analysis and will be used for evaluation of all endpoints. However, we acknowledge there may be attrition due to nurses leaving teams and where patients may be unavoidably switched to a nurse participant caseload in a group other than the one they were randomised to at baseline or to a MHN not in the study. To enable an evaluation of the effects of this on the primary outcome we will undertake and report a secondary per protocol [PP] analysis. HIP and non-HIP patients will be included in groups on an intention to treat basis. The primary analysis will include non-missing data only. However, where there is attrition at follow up the baseline characteristics of missing and non-missing values will be compared. Further, we will perform sensitivity analyses by (i) assuming zero change in scores and (ii) employing current multiple imputation techniques.

A per protocol analysis will also be performed. Per protocol will be defined as the participant remaining in the same arm of the study as at randomisation.

### Economic Analysis

This will assess whether HIP represents a cost-effective use of scarce NHS resources when compared to TAU. In line with guidance by NICE[47], costs will be calculated from the perspective of the NHS and personal social services and encompass those costs that are potentially related to the intervention in question. Thus we will monitor the levels of resource use associated with completing the HIP (including those associated with any associated tests/investigations that are recommended, changes in medication use and any other service referrals). For patients in both arms we will also monitor visits to other health care professionals, any admissions to hospital, and medication usage. Appropriate unit costs will subsequently be assigned to each of these items e.g. [46].

The measures of effectiveness employed in the economic analysis will be the 5 dimensions of the EQ-5D[48], used to calculate quality-adjusted-life-years (QALYs) associated with both the intervention and TAU.

An economic model will be constructed to estimate both the mean overall cost and mean overall effect associated with both the intervention and treatment as usual. If one of these options were shown to be less costly and more effective than the other then this would suggest that it 'dominates' the other, and represents a cost-effective use of scarce resources. Alternatively, the incremental cost-effectiveness ratio associated with the HIP will be estimated and assessed in relation to a set of cost-effectiveness thresholds e.g. a threshold of £20,000-£30,000 per QALY is recommended by NICE[47]. The associated level of uncertainty will also be characterised e.g. by estimating the cost-effectiveness acceptability curve [CEAC] for each intervention[49]. Additionally, sensitivity analysis will be undertaken to assess the robustness of conclusions to key assumptions.

### Cardiovascular risk

95% confidence intervals will be calculated for change in QRISK^©^2 for the HIP group participants.

### Process observation

Data from the HIP Audit Forms will be analysed using SPSS. Patient semi-structured interviews will be recorded by taking notes in the interview and seeking feedback from the patient participant that the notes accurately reflect what was said. The nurse focus groups and doctors' telephone interviews will be audio-recorded and transcribed verbatim. Transcripts from all interviews and focus groups will be coded using thematic analysis[50]. The results will then be returned to the participants for member checking[51].

### Potential Bias

The subjective nature of the self-report instruments, used for assessment, is accepted and every effort will be made to minimise potential bias which may occur due to this dynamic. In particular, patients may over or under report their health status depending on the trial arm to which they have been assigned. We acknowledge there may be attrition so to enable an evaluation of the effects of this on the primary outcome we will undertake a secondary per protocol[PP] analysis to compare with the ITT.

## Discussion

Physical comorbidities are common in people with SMI and have a profound effect on both quality and length of life. Poor physical health compounds the already major issue of social exclusion in this population. Preventing, recognising and treating physical comorbidity is central to holistic care and this is reflected in standards and guidelines from the Department of Health, the Nursing and Midwifery Council and the National Institute of Health and Clinical Excellence. Despite recent policy drivers to improve screening and intervention, concerns remain about the confidence and competence of the existing workforce to address this problem. Two studies, five years apart, highlighting this deficit in the MHN workforce in the UK have used observational designs in small convenience samples[26,52].

To date the only published experimental studies of implementation of a package of interventions to improve physical health in SMI have included a number of service evaluations and one RCT that aimed to improve screening rates [32,53-55]. With the exception of the HIP case series evaluation [32], these studies introduced new staff in a physical health role as an adjunct to the usual care delivered by the mental health team for the duration of a specific pilot project [53-55]. There remains a need to find a cost-effective evidence-based solution that avoids expensive and lengthy retraining or restructuring of services and roles and enables physical health screening and intervention on a population level.

In designing the HIP Programme, our focus has been to enable any MHN in contact with the person with SMI, to exploit the opportunity to monitor physical health and offer appropriate evidence-based or recommended intervention through their usual contact and role, rather than as an adjunct service. This is a pragmatic approach to the problem, yet it remains complex in terms of its evaluation and dissemination. Complex interventions contain several interacting components[56]. The HIP Programme targets the MHN, the patient and the organisation and requires them all to interact. For example, a MHN using the HIP with a patient will need to find time within their existing role to engage the patient in the HIP process and communicate within their own organisation and across the primary-secondary care interface to enable additional screening or access to intervention for the patient.

The Medical Research Council recommend researchers pay attention to the interplay between development, feasibility, evaluation and implementation when designing complex health interventions[56]. In the development stage, initial piloting of the HIP was positive with mental health nurses, patients, psychiatrists and GPs reporting that it is acceptable, avoids duplication of work, enables previously unidentified physical comorbidities to be uncovered and improves care planning and interface communication. Clinical utility and acceptability were then established in an exploratory case series within a community MH service in Scotland[32].

To maximise success in the future implementation of the HIP we have included an education package to meet the individual and organisational needs of the staff most likely to be in contact with people with SMI. The cluster design with the MHN as the cluster has been adopted because the focus of the intervention depends on the training and adoption of the HIP by MHNs. In evaluating the success of our intervention, the HIP and the training package together [the HIP Programme], we will evaluate patient outcomes and conduct a process observation. This will allow us to report education outcomes (any change in nurse knowledge of and attitudes towards a physical healthcare role using the adapted PHASe [40]) in addition to our primary evaluation of effectiveness and cost effectiveness.

The primary end point of this proposed study, change in physical related quality of life, has meaning to patients, carers, practitioners and policy makers and will formally explore the clinical potential of the tool. We have a specific qualitative element to explore patient, practitioner and medical experience of the HIP. Qualitative and audit data will enable us to more fully test the acceptability of this approach and further refine it as necessary. The health economic element of the study will provide important information about the costs of disseminating this approach across the NHS in terms of any additional service costs and Quality Adjusted Life Years (QALYs). The evaluation of HIP trainees knowledge and attitude towards physical health post HIP training will inform future workforce training and implementation plans.

If successful, this study will demonstrate the clinical and economic potential of an innovative brief training package and pragmatic clinical profile (the HIP Programme) that makes use of and builds upon the existing skills of MHNs who have the most contact with these patients and who already coordinate care and treatment between services. MHNs working in the community will be trained to profile and plan care around individual physical health issues in a real world setting. If successful, the quality of our evidence will inform regional dissemination and a future definitive effectiveness study to enable policy makers to recommend the HIP Programme across the NHS.

## Current Study Status

NHS ethics approval was granted in December 2010 and Research and Development Approval from the sponsor NHS Trust site in March 2011. Active recruitment commenced on 04 April 2011.

## List of abbreviations

CONSORT: Consolidated Standards of Reporting Trials; COPD: Chronic Obstructive Airways Disease; CRTU: Clinical Research Trials Unit; CVD: Cardiovascular Disease; DH: Department of Health; EQ-5D: EuroQuol Dimension Questionnaire; GP: General Practitioner; HIP: Serious Mental Illness Health Improvement Profile; HRU: Health Resource Use; ITT: Intention to Treat; MCQ: Multiple Choice Questionnaire; MCS: Mental Component Summary Score; MH: Mental Health; MHN: Mental Health Nurse; MHRN: Mental Health Research Network; MOS: Medical Outcome Study (see SF-36v2); NHS: National Health Service; NICE: National Institute of Health and Clinical Excellence; PHASe: Mental Health Nurse Physical Health Attitude Scale.

## Competing interests

JW has designed the HIP Cluster RCT as part of her PhD, supervised by RG.

## Authors' contributions

RG and JW conceived the study. JW (45%) wrote the paper under the direct supervision of RG (25%), LS (10%) and GB(10%) contributed the statistical and health economic methodology. MJ (10%) contributed details of the process observation methodology. All authors contributed to the draft of this manuscript for intellectual content and approved its final version.
